# Exposure to earthquakes and development of ischemic heart disease

**DOI:** 10.1186/s12889-024-17835-0

**Published:** 2024-02-12

**Authors:** Changwoo Han

**Affiliations:** Department of Preventive Medicine, Chungnam National University College of Medicine, 266, Munhwa-ro, Jung-gu, 35015 Daejeon, Korea

**Keywords:** Earthquake, Natural disaster, Ischemic heart disease, Difference-in-difference analysis

## Abstract

**Background:**

The evidence regarding the effect of earthquake exposure on the development of cardiovascular diseases is limited. This study evaluated the association between the 2016 Gyeongju earthquake, which had a magnitude of 5.8, and over 600 subsequent aftershocks occurring within a year in Korea, with the development of ischemic heart disease (IHD) among residents of Gyeongju.

**Methods:**

Ten years (2010–2019) of medical records from a randomly selected cohort of residents (*n* = 540,858) in Gyeongju and 3 control cities were acquired from the national health insurance service. Employing difference-in-difference and meta-analyses, the risks of IHD development of Gyeongju residents before (reference: Sep 2014 to Aug 2015; period 1: Sep 2015 to Aug 2016) and after (period 2: Sep 2016 to Aug 2017; period 3: Sep 2017 to Aug 2018; period 4: Sep 2018 to Aug 2019) the earthquake were estimated.

**Results:**

The monthly average incidence of IHD in Gyeongju was 39.5 persons (per 1,000,000) for reference period and 38.4 persons for period 1. However, the number increased to 58.5 persons in period 2, and 49.8 persons in period 3, following the earthquake. The relative risk (RR) [with a 95% confidence interval] of developing IHD among Gyeongju residents increased by 1.58 times (1.43, 1.73) in period 2, 1.33 times (1.21, 1.46) in period 3, and 1.15 times (1.04, 1.27) in period 4, in comparison to both the control cities and the pre-earthquake reference period. The increase in RR was particularly noticeable among women, adults aged 25–44, and individuals with lower incomes.

**Conclusions:**

The major earthquake in Korea was associated with an increase in the development of IHD among local residents. Individuals exposed to earthquakes may benefit from cardiovascular health surveillance.

**Supplementary Information:**

The online version contains supplementary material available at 10.1186/s12889-024-17835-0.

## Backgrounds

The Gyeongju earthquake, a seismic event with a moment magnitude of 5.8, occurred in Korea on September 12, 2016. This earthquake marked the most formidable seismic activity observed in Korean history [1, 2]. The estimated property loss was 10 million US dollars, and 632 aftershocks occurred over a year [3].

Disasters result in direct casualties and are known to affect mental health, especially in individuals who experience the loss of family, friends, and property [4]. Physical and mental stress following a disaster might lead to increased sympathetic tone and ultimately induce cardiovascular events [5]. Several review articles have reported increased rates of cardiovascular diseases and mortality in earthquake-affected regions [5–7].

Most previous studies on earthquakes and cardiovascular disease used time-series or cross-sectional study design by temporally comparing the number of hospital visits [8–10] or mortalities [11, 12] before and after the earthquake. Studies involving proper geographical controls have rarely been conducted [6, 13–15], and studies on the chronic effects of earthquakes on cardiovascular health using a cohort design were scarce [6, 8]. In addition, the lack of detailed personal medical histories in previous studies makes it difficult to elucidate the effects of earthquakes on new disease development [8, 16, 17].

The health insurance system of Korea registers all the hospital visit information of entire Koreans in a single database [18]. Earlier research employed the database to assess the immediate effects of an earthquake on the emergence of specific illnesses [3, 19]. Because hospital information is stored longitudinally, not only acute but also years of long-term effects of earthquakes and series of secondary shocks can be evaluated with the database.

Although the Gyeongju earthquake was the largest earthquake ever recorded in Korea, its magnitude and resulting damage were relatively small compared with those of the major earthquakes in history [3]. There were no direct casualties, and only 23 people were injured due to the earthquake [2]. However, its unexpected nature and the yearlong aftershocks might have led to mental and physical stress, ultimately affecting cardiovascular health [5, 6]. Therefore, it might be meaningful to evaluate whether earthquakes with limited casualties and property loss but long-lasting aftershocks can affect the cardiovascular health of residents exposed to the earthquake.

Therefore, this study examined the association between the 2016 Gyeongju earthquake and the risk of ischemic heart disease (IHD) development. The nationwide health insurance data was used to facilitate temporal and location-based comparisons of disease development.

## Materials and methods

### Study design

The difference-in-difference (DID) framework was used in this study [20]. If there were no external effects other than the earthquake, comparing the disease incidence rate before and after the earthquake-affected region (first difference) is considered a good estimate of the earthquake’s effect. To guard against the possibility of external effects, the same difference (second difference) in the control region (region not affected by the earthquake) was subtracted from the previous difference (first difference) to calculate the DID estimates.

**Fig. 1:**
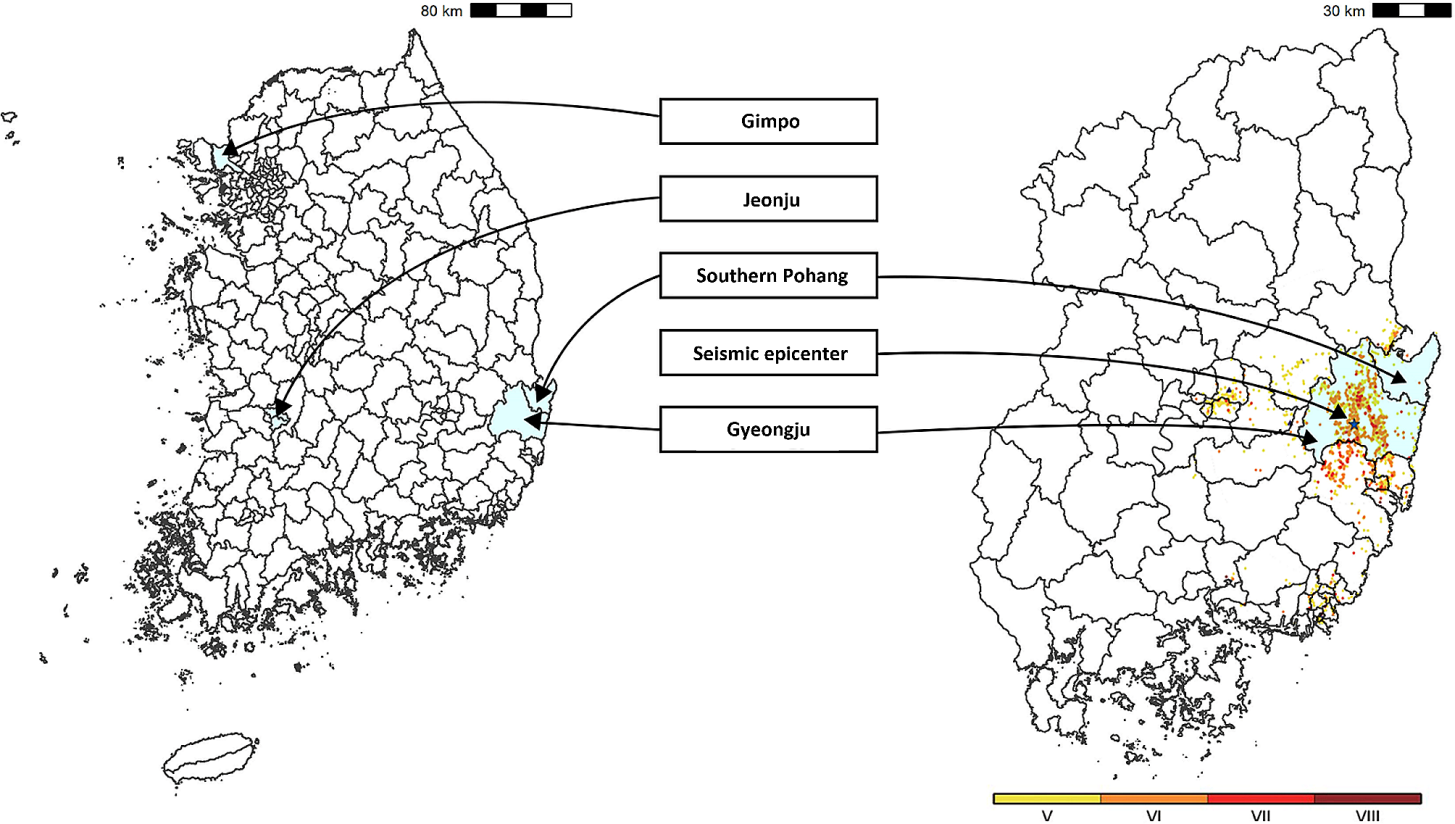
Map of the study region. Color-coded dots represent the extent of the Gyeongju earthquake exposure, calculated according to property damage, ranging from moment magnitude V (yellow) to VIII (red)

Considering the impact area of the Gyeongju earthquake (Fig. 1), residents in Gyeongju were identified as the earthquake-exposed group, while inhabitants of Gimpo, Jeonju, and Southern Pohang served as control groups, unaffected by the earthquake. Gimpo, with a population size comparable to Gyeongju, is situated 300 km away. Southern Pohang, being the nearest administrative division to Gyeongju, also shares a similar population size. Jeonju, larger in population than Gyeongju, lies approximately 186 km distant. The general characteristics, including the population size, ambient temperature, gross regional domestic product per capita, number of vehicles, number of hospitals, and the numbers of medical doctors per 1,000 population, for Gyeongju and the control cities have been extracted from the Korean Statistical Office and the Korea Meteorological Administration. Details are summarized in Table S1 (refer to Additional file 1). If there is a link between experiencing earthquakes and the development of IHD, it was anticipated that Gyeongju residents would demonstrate a higher occurrence of IHD after the earthquake, even when considering the temporal changes in the control cities.

### Data

South Korea operates a comprehensive single-payer health insurance system through the National Health Insurance Service (NHIS), offering healthcare coverage to the entire nation. For administrative purposes, hospital use information (including date, diagnostic code, details of treatment and prescription) and personal information (including birth year, sex, death, household health insurance premium, residential location) of the insured residents are archived in the National Health Insurance database [18].

The NHIS constructs a customized dataset based on researchers’ requests using data from the health insurance database. Prior epidemiological research utilized customized datasets to assess the impacts of air pollution, industrial operations, and natural calamities on public health [3, 19, 21, 22].

Due to the personal information protection policy, only a subset of entire population was allowed to be retrieved from the National Health Insurance database. For this study, 50% of individuals who resided in Gyeongju, Gimpo, Southern Pohang, and Jeonju in 2014 were randomly selected (*n* = 732,554). Sampling was conducted by the NHIS statistical service team based on sex and birth year. To avoid population migration-related problems [23], individuals who did not change their place of residence from 2014 to 2019 or from 2014 to death were selected (*n* = 540,858). The records of the individuals included in the study sample were screened for all IHD-related hospital visits using primary diagnostic code information of each hospital visits (*International Classification of Disease 10th revision codes, I20.X to I25.X)*. From 2010 to 2019, 460,582 hospital visits for IHD were identified. The incidence was calculated based on the first hospital visit in which diagnostic codes were recorded as in previous studies [24]. The monthly number of patients newly diagnosed with IHD was calculated for various age brackets, spanning five-year ranges from 0 to 4 to 80–84 years and those aged 85 years and above. The monthly age-standardized incidence rate was calculated to determine the overall IHD incidence patterns during the study period and to compare the patterns across the evaluated cities with different populations and age structures [25, 26]. The number of the study population by each city is presented in Supplementary Table S2 (see Additional file 1). The regional ambient temperature data used in the sensitivity analysis was obtained from the Korea Meteorological Administration’s Automated Synoptic Observing System.

### Statistical analysis

The following DID model was used:

$$\begin{aligned} \log {\mu _{t,x}} & = {\beta _0} + \beta_1R + \beta_2ns\left( {{T_t},df = 6} \right) \\ & \quad + \beta_3{P_1} + \beta_{4}{P_2} + \beta_5{P_3} + \beta_6{P_4} \\ & \quad + \beta_7R\,{P_1} + \beta_8R\,{P_2} \\ & \quad + \beta_9R\,{P_3} + \beta_{10}R\,{P_4} \\ & \quad + \beta_{11}M + \beta_{12}{\text{A}} \\ & \quad + {\text{offset}}\left( {\log Po{p_{t,x}}} \right) + \varepsilon \\ \end{aligned}$$

In this model, $$ {\mu }_{t, x} $$represents the number of IHD incidence patients in year-month *t* and age group *x*, $$ R$$ represents the study region (*R* = 1: Gyeongju city, *R* = 0: control city), and $$ {T}_{t}$$ represents the time (in months) since Sep 2014. $$ {P}_{n}$$ represents the period before and after the Gyeongju earthquake ($$ {P}_{1} $$= Sep 2015-Aug 2016, $$ {P}_{2} $$= Sep 2016-Aug 2017, $$ {P}_{3} $$= Sep 2017-Aug 2018, $$ {P}_{4} $$= Sep 2018-Aug 2019), M refers to months, A represents age categories, and$$ { Pop}_{t, x }$$ represents the population qualified for health insurance within the specific age group *x* and during the *t* year-month. A natural spline term for the time variable was utilized to account for long term temporal patterns in IHD incidence over time, as indicated by the variable $$ ns ({T}_{t}, df=6)$$. Month indicator variable (*M*) and age category variable (*A*) were included to adjust seasonality and age structure. The $$ {{\upbeta }}_{7}$$ to $$ {{\upbeta }}_{10}$$ values were used to calculate the risk of disease development in Gyeongju residents in each time period by setting the period from Sep 2014 to Aug 2015 as a reference. The estimates between Gyeongju and the control cities were meta-analyzed using a random-effects model in order to calculate the pooled estimates [27]. A similar DID model was used in a previous study that evaluated the mental health effects of an induced earthquake in Korea [19].

**Fig. 2:**
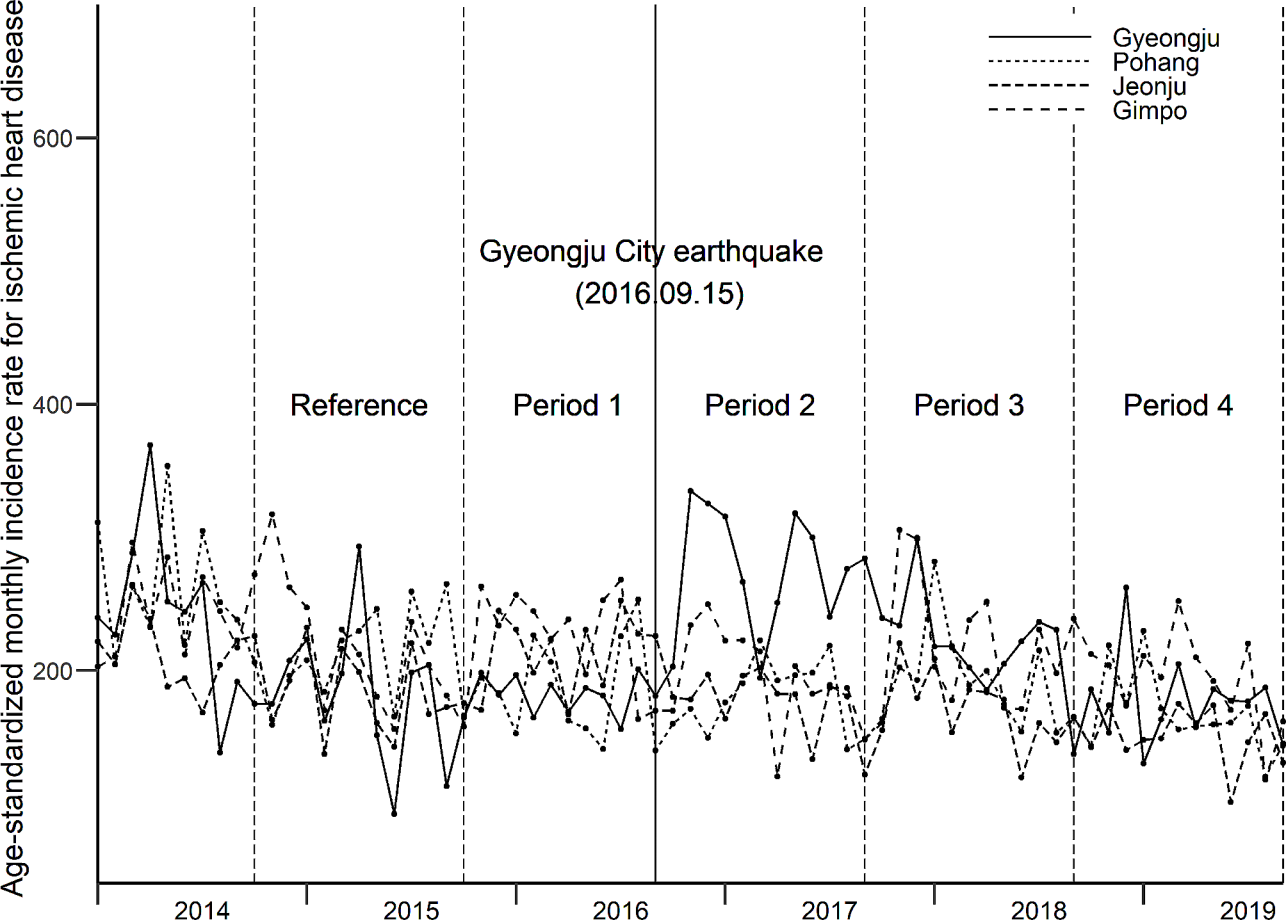
Monthly age-standardized ischemic heart disease incidence rate (1,000,000 persons)

To confirm the parallel-trend assumption in the DID analysis, monthly age-standardized incidence rates in the analyzed cities were visually inspected (Fig. 2). In addition, the reference (Sep 2014-Aug 2015) and $$ {P}_{1}$$ (Sep 2015-Aug 2016) periods before the earthquake were compared with the expectation of null results. To show that the increase in disease development was confined to Gyeongju, comparisons were performed between the control cities.

A stratification analysis was conducted based on gender, age categories (25–44, 45–64, and 65 and above), and income groups and potential effect modification was evaluated with the Cochrane Q test [28]. Patients aged < 24 years were excluded from the stratification analysis because only a small number developed IHD. Age categories (25–44, 45–64, and 65 and above) were selected based on previous cardiovascular epidemiological studies [29, 30]. Health insurance premiums (categorized from 1 to 20) vary according to household income in Korea. As in previous studies, income groups were defined by using the categorized health insurance premium levels (low: 0 to 10, high: 11–20) [21]. For the sensitivity analysis, monthly average temperature for each region was included in the difference-in-difference model to adjust for region specific climate differences. SAS (version 9.4) and R statistical software (version 4.2.1) were used in our analysis. The significance level for statistical analysis was established as *p* < 0.05.

## Results

Ten years (2010–2019) of medical records from a randomly selected cohort of residents (*n* = 540,858) in Gyeongju and 3 control cities were evaluated. A total of 11,039 newly diagnosed IHD patients were identified between September 2014 and August 2019.

Table 1; Fig. 2 show the monthly IHD incidence in Gyeongju and in the control cities before (reference: Sep 2014-Aug 2015, period 1: Sep 2015-Aug 2016) and after the earthquake (period 2: Sep 2016-Aug 2017, period 3: Sep 2017-Aug 2018, period 4: Sep 2018-Aug 2019). A monthly average incidence of IHD in Gyeongju was 39.5 (per 1,000,000) for reference and 38.4 for period 1. However, this number increased to 58.5 in period 2, and 49.8 in period 3, following the earthquake. There was a clear increase in the age-standardized incidence rate (/1,000,000 persons) in Gyeongju city after the earthquake (period 2: 267.1 persons, period 3: 231.2 persons/1,000,000 persons), while no difference was observed in the control cities.

The relative risk [RR, 95% confidence interval (CI)] of developing IHD among Gyeongju residents increased by 1.58 times (1.43, 1.73) in period 2, 1.33 times (1.21, 1.46) in period 3, and 1.15 times (1.04, 1.27) in period 4, in comparison to both the control cities and the pre-earthquake reference period (Table 1). As expected, no increase was observed before the earthquake (period 1) [RR (95% CI): 0.97 (0.86, 1.11)]. Figure 2 also shows similar monthly disease incidence patterns across the cities before the earthquake.

A monthly average incidence of IHD in Gyeongju was 21.3 (per 1,000,000; reference) and 19.8 (period 1) in men and 18.2 (reference) and 18.7 (period 1) in women. However, this number increased to 29.2 and 29.3 after the earthquake in both men and women (period 2). The increase was greater in women [RR (95% CI): men, 1.38 (1.20, 1.60); women, 1.85 (1.60, 2.13), p-value for heterogeneity: < 0.01] (Table S3, see Additional file 1).

Table 2 presents the results of the stratification analysis by age and income groups. A monthly average incidence of IHD in Gyeongju for reference period was 1.9, 15.5, and 21.7 (per 1,000,000) in age 25–44, age 45–64, and age 65- group, respectively. However, this number increased to 2.8 (age 25–44), 24.6 (age 45–64), and 30.8 (age 65-) after the earthquake (period 2). The DID estimates for IHD development were greater in young adults (25–44 years) than in other age groups (45–64 and ≥ 65 years) [RR (95% CI) for period 2: age 25–44, 2.36 (1.50, 3.73); age 45–64, 1.65 (1.42, 1.91); age ≥ 65, 1.48 (1.25, 1.74): p-value for heterogeneity: 0.05] (Table S4, see Additional file 1).

A monthly average incidence of IHD in Gyeongju was 13.9 (per 1,000,000; reference) and 14.2 (period 1) in low income group and 25.6 (reference) and 24.3 (period 1) in high income group. However, this number increased to 23.8 and 34.7 in both low- and high-income groups after the earthquake (period 2) (Table 2). Although the risk of IHD development was greater in the low-income group than in the high-income group [RR (95% CI) for period 2: low-income group, 1.77 (1.52, 2.07); high-income group: 1.47 (1.31, 1.65)], the difference was marginally significant (p-value for heterogeneity: 0.06) (Table S5, see Additional file 1). The DID estimates between the control cities are shown in Supplementary Table S6 (see Additional file 1). No increase was observed across the control cities during the study period. The sensitivity analysis additionally adjusting for monthly average ambient temperature showed similar results to the main analysis (Supplementary Table S7, see Additional file 1).

**Table 1:** The ischemic heart disease incidence counts and rates before and after the 2016 Gyeongju earthquake

	Monthly average incidence of IHD patients (N)				Age standardized monthly average incidence rate (/1,000,000 person)				Difference-in-difference estimates (95% confidence interval)			
	Gyeongju	Pohang	Gimpo	Jeonju	Gyeongju	Pohang	Gimpo	Jeonju	Gyeongju vs. Pohang	Gyeongju vs. Gimpo	Gyeongju vs. Jeonju	Pooled estimates
Total												
2014.09-2015.08	39.5	34.1	38.6	68.8	189.2	211.2	225.4	189.8				
2015.09-2016.08	38.4	30.6	39.0	76.9	175.1	194.3	223.3	206.7	1.10 (0.91, 1.32)	0.98 (0.82, 1.16)	0.88 (0.75, 1.02)	0.97 (0.86, 1.11)
2016.09-2017.08	58.5	29.9	37.2	70.5	267.1	179.6	196.8	184.2	1.73 (1.44, 2.07)^a^	1.58 (1.34, 1.88)^a^	1.48 (1.28, 1.71)^a^	1.58 (1.43, 1.73)^a^
2017.09-2018.08	49.8	32.8	39.6	66.0	231.2	190.9	212.8	167.6	1.36 (1.13, 1.63)^a^	1.28 (1.08, 1.52)^a^	1.35 (1.17, 1.57)^a^	1.33 (1.21, 1.46)^a^
2018.09-2019.08	39.8	30.0	37.8	62.3	176.7	169.2	199.6	153.3	1.20 (0.99, 1.45)	1.09 (0.91, 1.30)	1.16 (0.99, 1.35)	1.15 (1.04, 1.27)^a^
Men												
2014.09-2015.08	21.3	18.8	21.4	37.1	226.7	239.6	266.2	227.3				
2015.09-2016.08	19.8	18.8	22.3	44.8	206.3	258.6	270.5	266.9	0.93 (0.74, 1.17)	0.90 (0.71, 1.13)	0.77 (0.63, 0.95)	0.86 (0.75, 0.98)
2016.09-2017.08	29.2	16.5	21.5	41.8	307.8	215.5	239.5	242.0	1.58 (1.26, 1.98)^a^	1.40 (1.12, 1.74)^a^	1.23 (1.01, 1.50)^a^	1.38 (1.20, 1.60)^a^
2017.09-2018.08	28.5	19.9	22.5	39.3	305.2	243.1	258.5	222.7	1.29 (1.04, 1.61)^a^	1.32 (1.06, 1.65)^a^	1.29 (1.06, 1.57)^a^	1.30 (1.15, 1.47)^a^
2018.09-2019.08	22.0	17.3	21.8	36.6	219.7	212.1	245.2	200.4	1.16 (0.92, 1.46)	1.06 (0.84, 1.33)	1.08 (0.88, 1.32)	1.10 (0.97, 1.24)
Women												
2014.09-2015.08	18.2	15.3	17.2	31.7	151.7	178.9	186.8	155.6				
2015.09-2016.08	18.7	11.8	16.7	32.2	144.3	137.2	179.0	152.6	1.36 (1.02, 1.82)^a^	1.08 (0.83, 1.40)	1.03 (0.81, 1.30)	1.13 (0.97, 1.31)
2016.09-2017.08	29.3	13.4	15.7	28.7	228.2	146.0	156.7	131.4	1.90 (1.45, 2.49)^a^	1.83 (1.42, 2.35)^a^	1.83 (1.46, 2.30)^a^	1.85 (1.60, 2.13)^a^
2017.09-2018.08	21.3	12.8	17.1	26.7	161.1	139.5	169.0	119.8	1.46 (1.10, 1.93)^a^	1.24 (0.96, 1.60)	1.45 (1.14, 1.83)^a^	1.38 (1.19, 1.60)^a^
2018.09-2019.08	17.8	12.8	16.0	25.7	136.0	127.7	157.5	111.3	1.25 (0.93, 1.66)	1.12 (0.86, 1.46)	1.27 (1.00, 1.62)^a^	1.21 (1.04, 1.41)^a^

^a^P-value < 0.05

**Table 2:** The ischemic heart disease incidence counts and rates before and after the 2016 Gyeongju earthquake (analyses by age and income groups)

	Monthly average incidence of IHD patients (N)				Age standardized monthly average incidence rate (/1,000,000 person)				Difference-in-difference estimates (95% confidence interval)			
	Gyeongju	Pohang	Gimpo	Jeonju	Gyeongju	Pohang	Gimpo	Jeonju	Gyeongju vs. Pohang	Gyeongju vs. Gimpo	Gyeongju vs. Jeonju	Pooled estimates
Age 25–44												
2014.09-2015.08	1.9	2.1	3.8	5.0	114.6	132.0	154.4	106.2				
2015.09-2016.08	1.9	2.1	4.2	5.0	102.8	136.0	169.8	134.1	1.00 (0.46, 2.17)	0.93 (0.43, 1.99)	1.00 (0.48, 2.12)	0.98 (0.63, 1.52)
2016.09-2017.08	2.8	1.7	1.4	3.8	214.9	119.9	65.4	92.7	1.86 (0.87, 3.99)	4.06 (1.77, 9.33)^a^	1.94 (0.95, 3.98)	2.36 (1.50, 3.73)^a^
2017.09-2018.08	2.9	1.6	3.3	3.9	159.9	136.1	122.8	111.3	2.03 (0.94, 4.36)	1.83 (0.88, 3.81)	1.98 (0.97, 4.03)	1.94 (1.27, 2.97)^a^
2018.09-2019.08	2.1	1.8	3.5	3.4	123.9	131.5	156.0	90.7	1.31 (0.60, 2.87)	1.21 (0.56, 2.59)	1.63 (0.76, 3.49)	1.37 (0.88, 2.14)
Age 45–64												
2014.09-2015.08	15.5	17.0	16.3	29.3	632.7	824.9	614.7	590.8				
2015.09-2016.08	15.9	15.7	16.4	35.2	747.3	772.0	594.6	633.4	1.11 (0.86, 1.44)	1.05 (0.77, 1.42)	0.86 (0.67, 1.12)	1.00 (0.85, 1.17)
2016.09-2017.08	24.6	14.8	16.7	31.8	1065.5	747.3	616.8	606.1	1.83 (1.42, 2.34)^a^	1.64 (1.22, 2.19)^a^	1.50 (1.17, 1.91)^a^	1.65 (1.42, 1.91)^a^
2017.09-2018.08	20.2	17.6	17.8	28.2	858.1	961.3	691.3	562.7	1.25 (0.98, 1.61)	1.29 (0.96, 1.73)	1.40 (1.09, 1.81)^a^	1.31 (1.13, 1.53)^a^
2018.09-2019.08	14.9	14.8	16.6	28.2	655.9	830.4	604.8	503.2	1.10 (0.85, 1.43)	1.04 (0.77, 1.42)	1.05 (0.80, 1.37)	1.07 (0.91, 1.25)
Age 65-												
2014.09-2015.08	21.7	15.0	18.3	34.0	1255.7	1240.7	1365.9	1122.1				
2015.09-2016.08	20.6	12.6	18.2	36.1	1128.7	955.2	1317.5	1164.1	1.15 (0.83, 1.61)	0.96 (0.70, 1.30)	0.90 (0.68, 1.18)	0.98 (0.82, 1.17)
2016.09-2017.08	30.8	13.3	19.1	33.9	1725.9	999.4	1338.8	1018.7	1.67 (1.22, 2.29)^a^	1.38 (1.03, 1.85)^a^	1.43 (1.10, 1.86)^a^	1.48 (1.25, 1.74)^a^
2017.09-2018.08	26.3	13.5	18.3	33.3	1501.3	1075.9	1190.3	1019.4	1.44 (1.05, 1.98)^a^	1.25 (0.93, 1.69)	1.26 (0.96, 1.64)	1.30 (1.10, 1.54)^a^
2018.09-2019.08	22.5	13.5	17.1	30.4	1094.7	1005.7	1106.6	907.6	1.26 (0.91, 1.75)	1.16 (0.85, 1.57)	1.18 (0.90, 1.56)	1.20 (1.00, 1.42)^a^
Low income												
2014.09-2015.08	13.9	12.8	14.7	27.3	185.7	229.6	215.6	189.3				
2015.09-2016.08	14.2	10.9	15.0	29.6	188.1	198.2	219.3	198.8	1.20 (0.89, 1.62)	1.01 (0.76, 1.34)	0.95 (0.74, 1.22)	1.03 (0.88, 1.21)
2016.09-2017.08	23.8	10.4	15.3	29.3	299.4	187.1	205.1	190.2	2.13 (1.60, 2.84)^a^	1.69 (1.29, 2.21)^a^	1.63 (1.30, 2.06)^a^	1.77 (1.52, 2.07)^a^
2017.09-2018.08	17.4	13	15	28.6	219.4	220	205.7	182.5	1.25 (0.94, 1.67)	1.27 (0.96, 1.67)	1.24 (0.97, 1.58)	1.25 (1.07, 1.46)^a^
2018.09-2019.08	15.2	10.6	15.3	24.8	192.7	171.1	206.9	150.8	1.35 (1.00, 1.82)^a^	1.09 (0.82, 1.44)	1.26 (0.98, 1.62)	1.23 (1.05, 1.44)^a^
High income												
2014.09-2015.08	25.6	21.3	23.9	41.5	191.4	201.6	234.7	191.0				
2015.09-2016.08	24.3	19.7	24.0	47.3	166.7	195.4	224.7	215.2	1.04 (0.83, 1.30)	0.96 (0.77, 1.19)	0.84 (0.69, 1.01)	0.93 (0.82, 1.06)
2016.09-2017.08	34.7	19.5	21.9	41.2	247.3	176.0	190.3	181.7	1.52 (1.23, 1.89)	1.53 (1.24, 1.88)	1.39 (1.16, 1.67)	1.47 (1.31, 1.65)^a^
2017.09-2018.08	32.3	19.8	24.6	37.4	235.7	176.0	217.0	153.7	1.42 (1.15, 1.77)	1.29 (1.05, 1.59)	1.44 (1.19, 1.74)	1.38 (1.23, 1.56)^a^
2018.09-2019.08	24.6	19.4	22.5	37.5	167.3	168.9	197.9	155.2	1.12 (0.89, 1.40)	1.09 (0.88, 1.35)	1.10 (0.91, 1.34)	1.10 (0.98, 1.25)

^a^P-value < 0.05

## Discussion

The residents affected by the earthquake showed an increased risk of IHD development. The risk of IHD development in Gyeongju residents increased by 50% during the year following the earthquake compared to residents in cities unaffected by the earthquake. This increase in risk was particularly noticeable among women, adults aged 25–44, and individuals with lower incomes. Based on the study findings, close monitoring of long-term cardiovascular health is recommended for earthquake-exposed residents.

Several research studies have documented a connection between the earthquake and cardiovascular health [5–7]. For instance, an increase in cardiac-related deaths and IHD admissions was reported on the day or within a week after the 1994 Northridge earthquake (magnitude: 6.7) in the US [10–12]. A rise in hospital admissions due to acute myocardial infarction and cardiomyopathy was observed five weeks after a sequence of major earthquakes took place in Christchurch, New Zealand, in 2010 (magnitude: 7.1) and 2011 (magnitude: 6.3) [16]. Research utilizing medical records demonstrated an increased risk of cardiovascular events occurring within a span of three weeks following the Great East Japan Earthquake in 2011, which had a magnitude of 9.0 [8, 9].

The long-term consequences of earthquakes on the cardiovascular health of residents have received limited attention in earlier studies [6]. An increase mortality due to cardiovascular disease and in the development of other morbidities (hypertension, heart disease, diabetes, and arthritis) were reported in a cohort study involving residents exposed to the 1988 Armenia earthquake, which had a magnitude of 6.8 [15]. Comparing the 5 years preceding and the 3 years following the Niigata-Chuetsu earthquake in Japan (magnitude: 6.8) in 2004, a 14% rise in acute myocardial infarction-related mortality was noted after the earthquake [13].

Within 1 year after the Great East Japan earthquake, an increase in out-of-hospital cardiac arrest cases was observed among residents who suffered significant earthquake damage [17]. A rise in fatal myocardial infarction occurrences was identified in the area impacted by the tsunami triggered by the Great East Japan earthquake, and this increase persisted for 3 years [14]. However, when studying a group of heart failure patients, stable mortality patterns were observed throughout the 3-year monitoring period after the Great East Japan earthquake, even though there was a notable surge shortly after the earthquake [31].

Various epidemiological studies report that earthquakes affect not only cardiovascular diseases but also infectious and respiratory diseases and psychological disorders [6, 32–34]. A systematic literature review of 160 documents evaluating the health impact of the Great East Japan Earthquake in 2011 found that medical utilization increased for patients with pre-existing respiratory and mental disorders after the earthquake [32]. Additionally, there was a rise in the incidence of new mental disorders, including post-traumatic stress disorder, suicidal tendencies, and depression, among residents in earthquake-affected areas [32].

Considering the anticipated increase in earthquake intensity and frequency due to the impact of climate change in regions such as Korea [35], it is crucial to quantitatively assess not only the direct injuries and fatalities resulting from earthquakes but also the diverse health impact on community residents using the well-designed epidemiological studies. However, most of previous studies use cross-sectional and questionnaire based evaluation for a small number of participants without using temporal or geographical location based compaisons [6, 32–34]. In addition, major earthquakes with high number of death tolls were mainly considered.

This study focusing on 2016 Gyeongju earthquake and series of aftershocks used both temporal and geographical control groups based on the health insurance database, and the findings suggest an association between earthquake exposure and an increase in IHD incidence. Disease incidence after an earthquake can be determined without bias only if a community-based cohort already existed at the earthquake site [31, 36, 37]. Because an earthquake occurs randomly, the possibility of a such pre-existing cohort is very low. Therefore, a previous study recruited subjects after an earthquake and investigated previous histories and new disease onset using a structured questionnaire [15]. In contrast, this research managed to determine the connection between earthquake exposure and disease occurrence post-earthquake by examining the medical records of residents through hospital visit data. Utilizing hospital-based or health insurance data, which often include comprehensive disease outcomes, enables the assessment of the diverse impact of earthquakes on different disease [3, 19].

Physical and emotional stress caused by earthquake exposure affects the sympathetic nervous and endocrine systems, which may precipitate cardiovascular events [5, 6]. The experience of life-threatening events or the loss of family or close friends during such an event may be the main drivers of emotional stress [4]. Reports indicate that increased psychological stress levels or biomarkers, such as glycated hemoglobin, have lasted for a year after earthquake exposure [5, 6]. Studies in Korea showed an increase in anxiety-related disorders and mood disorders within the days and weeks after the earthquake [3, 19]. Therefore, the sudden earthquake experience and yearlong aftershocks may have affected the cardiovascular health of Gyeongju residents.

Another plausible mechanism explaining the increased risk of cardiovascular disease after a disaster is an interruption in medical services [9]. Loss of medications or destruction of medical facilities after a disaster may cause such interruptions. However, the estimated property damage was relatively small after the Gyeongju earthquake compared to the damage caused by major earthquakes in previous studies [3]. In addition, as far as author is aware, no disruption of medical services was reported in Gyeongju after the earthquake. Therefore, the effects of healthcare disruption on the findings of this study may be minimal.

Occupational studies have shown an association between whole-body vibration and cardiovascular disease [38]. Vibration exposure is associated with vasoconstriction, endothelial dysfunction, and increased heart rate and blood pressure [38]. An experimental study showed that exposure to simulated train vibration increased heart rate [39]. Although over 96% of the aftershocks following the Gyeongju earthquake were below a magnitude of 3 (most people inside buildings were not able to feel the vibration) [40], chronic long-term minor exposure to sudden vibration may have affected the residents’ cardiovascular health. However, additional laboratory and epidemiological evidence is required to confirm this hypothesis.

Studies focusing on a vulnerable population against the effects of earthquake on IHD incidence are still limited. In this study, subjects from the low household income group and women exhibited an increased risk of IHD development after the earthquake. Although the biological mechanisms are unclear, women and individuals from low-income groups exhibit a heightened risk of adverse health effects after disasters [3, 41, 42]. A prospective cohort study conducted in Japan revealed an elevated risk of cardiovascular deaths in women who reported high perceived stress compared to those in the low-stress, while the association was found to be less pronounced among men [43]. Subjects with a low socioeconomic status may have a vulnerable health status or infrastructure, which can make them susceptible to external stresses [44, 45]. Similar findings were noted in previous studies investigating the health effects of earthquakes. The standardized incident ratio of fatal myocardial infarction comparing the incidence before (2009–2010) and after 2011 Great East Japan earthquake showed higher rates in women after the earthquake (2011: 1.72; 2012; 2.59) than men (2011: 1.85; 2012; 1.58) [14]. By comparing acute myocardial infarction mortality rate 5 years before and 3 years after the Niigata-Chuetsu earthquake, the increase was greater in women (14.9% increase after the earthquake) compared to men (13.4% increase after the earthquake) [13]. Risk of developing heart disease after the 1988 Armenia earthquake was 1.5 times higher in low education group (education years ≤ 10 years) compared to high education group in gender matched case-control analysis [15]. It is important to identify vulnerable groups to natural disasters in disaster research studies. Based on such studies, it will be possible to determine priority targets for the healthcare management of disaster victims [46].

The results of age stratification analysis showed a higher estimate for the age 25–44 group compared to the age 45–64 and age ≥ 65 groups during the year following the Gyeongju earthquake. However, the results from 25 to 44 age group should be carefully interpreted due to the low monthly incidence rate of IHD (ranging from 1.9 to 5.0 persons per month) in both the Gyeongju and control regions. In addition, previous earthquake studies comparing the effects of earthquake across different age categories did not show significant differences [14, 15].

This study has several limitations. First, the validity of the DID analysis can be threatened by external factors specifically affecting Gyeongju. The DID analysis may not separate the effects of the earthquake and other external factors changing in Gyeongju at the same time as the earthquake. Although such changes were not noticed, the possibility cannot be ruled out. Second, individual factors other than sex, age, and household income were not considered in this study because of data availability. The availability of information regarding routine health examinations or personal habits could have enabled individual-level matching, which may have increased the comparability between the case (individuals from Gyeongju) and control (individuals from the control cities) groups compared to the geographical and stratification-based analyses used in this study. Third, specific subtypes of IHD or assessments for other cardiovascular diseases were not feasible due to data constraints. In addition, due to the personal information protection policy, only 50% of entire population was evaluated in this study. Further analysis including detailed assessments of disease subtypes and the entire population will help to shape overall disease burden of 2016 Gyeongju earthquake on residents.

## Conclusions

Analyzing the nationwide health insurance data showed a marked increase in the incidence of IHD in earthquake-exposed residents. The DID analysis showed a 58% increase in the risk of IHD development, and this increased risk was found to last for over 2 years. Although the damage to life and property was limited after the Gyeongju earthquake, this study revealed an association between long-lasting seismic activities and the development of IHD.

### Electronic supplementary material

Below is the link to the electronic supplementary material.

Supplementary Material 1

## Data Availability

The data that support the findings of this study are available from the National Health Insurance Service, Korea. Restrictions apply to the availability of these data, which were used under license for this study. Data are available from the authors (contact C.H.) with the permission of the National Health Insurance Service, Korea.

## Declarations

### Statement on guidelines

This study complies with relevant guidelines and regulations. All the data were provided by the National Health Insurance Service (NHIS). This study used the de-identified data provided by the Statistics Korea and the NHIS.
