# Supplementary material for: Exposure to earthquakes and development of ischemic heart disease
Source: BMC Public Health. 2024 Feb 12;24:446. doi: 10.1186/s12889-024-17835-0 (PMC10863258; doi:10.1186/s12889-024-17835-0)
Supplement: Supplementary file 1 — Supplementary Material 1 [file 12889_2024_17835_MOESM1_ESM.docx]

Additional file 1

Table of contents

Table S1. General information of Gyeongju, Gimpo, Jeonju, and Southern Pohang (for year 2016).

Table S2. Yearly number of study subjects

Table S3. The difference between pooled estimates for gender stratified estimation

Table S4. The difference between pooled estimates for age group stratified estimation

Table S5. The difference between pooled estimates for income group stratified estimation

Table S6. Association between the Gyeongju earthquake and changes in the monthly incidence of ischemic heart disease patients in control cities.

Table S7. The difference-in-difference estimates with additional adjustment for monthly average temperature

Table S1. General information of Gyeongju, Gimpo, Jeonju, and Southern Pohang (for year 2016).

|  | Gyeongju | Gimpo | Jeonju | Southern Pohang |
| --- | --- | --- | --- | --- |
| Area (km^2^) | 1,324 | 276.6 | 205.5 | 393 |
| Average annual temperature (℃) | 13.3 | 12.2 | 12.8 | 14.4 |
| Midyear registered residents (n) |  |  |  |  |
| Total | 257,733 | 354,626 | 648,854 | 244,037 |
| Less than 25 years, n(%) | 61,872 (24.0) | 103,913(29.3) | 196,355 (30.3) | 68,899 (26.2) |
| 25 to 64 years, n(%) | 148,188 (57.5) | 211,734 (59.7) | 372,835 (57.5) | 149.601 (60.6) |
| Equal to or more than 65 years of age, n(%) | 47,674 (18.5) | 38,980 (11.0) | 79,665 (12.3) | 30,538 (12.5) |
| Gross regional domestic product per capita (1,000 Korean Won) | 10,788 | 11,420 | 13,886 | 8,416^a^ |
| Number of vehicles | 97,769 | 119,591 | 237,868 | 93,508 |
| Number of hospitals | 394 | 498 | 1314 | 343 |
| Number of medical doctors per 1,000 population | 2.0 | 1.7 | 3.7 | 2.2^b^ |

^a^ Divide the values of total Pohang with 2 (Southern and Northern Pohang)

^b^ Values for Pohang was used

Table S2. Yearly number of study subjects

| City | Men | Women | 25-44 | 45-64 | 65- | Low income | High income |
| --- | --- | --- | --- | --- | --- | --- | --- |
| Gyeongju | 50495 (49.3) | 51985 (50.7) | 28396 (27.7) | 33360 (32.6) | 16538 (16.1) | 39141 (38.2) | 63339 (61.8) |
| Gyeongju | 51023 (49.3) | 52468 (50.7) | 27310 (26.4) | 34337 (33.2) | 17452 (16.9) | 39533 (38.2) | 63958 (61.8) |
| Gyeongju | 51532 (49.3) | 52963 (50.7) | 26230 (25.1) | 34941 (33.4) | 18779 (18.0) | 39923 (38.2) | 64572 (61.8) |
| Gyeongju | 51983 (49.4) | 53351 (50.6) | 24919 (23.7) | 35660 (33.9) | 20152 (19.1) | 40258 (38.2) | 65076 (61.8) |
| Gyeongju | 52423 (49.4) | 53742 (50.6) | 23898 (22.5) | 36141 (34.0) | 21587 (20.3) | 40585 (38.2) | 65580 (61.8) |
| Gyeongju | 51905 (49.4) | 53246 (50.6) | 22718 (21.6) | 36575 (34.8) | 22071 (21.0) | 40164 (38.2) | 64987 (61.8) |
| Gyeongju | 51408 (49.3) | 52789 (50.7) | 21721 (20.8) | 36900 (35.4) | 22650 (21.7) | 39757 (38.2) | 64440 (61.8) |
| Gyeongju | 50909 (49.3) | 52332 (50.7) | 20823 (20.2) | 37006 (35.8) | 23422 (22.7) | 39332 (38.1) | 63909 (61.9) |
| Gyeongju | 50444 (49.3) | 51889 (50.7) | 20027 (19.6) | 37159 (36.3) | 24123 (23.6) | 38992 (38.1) | 63341 (61.9) |
| Gyeongju | 49973 (49.3) | 51402 (50.7) | 19452 (19.2) | 37115 (36.6) | 24871 (24.5) | 38613 (38.1) | 62762 (61.9) |
| Gimpo | 54741 (49.9) | 54996 (50.1) | 37354 (34.0) | 29490 (26.9) | 10244 (9.3) | 41758 (38.1) | 67979 (61.9) |
| Gimpo | 55710 (50.0) | 55811 (50.0) | 36382 (32.6) | 30664 (27.5) | 11149 (10.0) | 42493 (38.1) | 69028 (61.9) |
| Gimpo | 56718 (50.0) | 56647 (50.0) | 35349 (31.2) | 31765 (28.0) | 12206 (10.8) | 43286 (38.2) | 70079 (61.8) |
| Gimpo | 57564 (50.1) | 57283 (49.9) | 34258 (29.8) | 32978 (28.7) | 13267 (11.6) | 43967 (38.3) | 70880 (61.7) |
| Gimpo | 58341 (50.2) | 57967 (49.8) | 33018 (28.4) | 34319 (29.5) | 14357 (12.3) | 44607 (38.4) | 71701 (61.6) |
| Gimpo | 57976 (50.1) | 57715 (49.9) | 31678 (27.4) | 35524 (30.7) | 14873 (12.9) | 44322 (38.3) | 71369 (61.7) |
| Gimpo | 57682 (50.1) | 57435 (49.9) | 30482 (26.5) | 36940 (32.1) | 15256 (13.3) | 44080 (38.3) | 71037 (61.7) |
| Gimpo | 57345 (50.1) | 57174 (49.9) | 29346 (25.6) | 37891 (33.1) | 16092 (14.1) | 43808 (38.3) | 70711 (61.7) |
| Gimpo | 57031 (50.1) | 56904 (49.9) | 28351 (24.9) | 38932 (34.2) | 16772 (14.7) | 43550 (38.2) | 70385 (61.8) |
| Gimpo | 56693 (50.0) | 56649 (50.0) | 27420 (24.2) | 39852 (35.2) | 17556 (15.5) | 43289 (38.2) | 70053 (61.8) |
| Jeonju | 106624 (48.4) | 113641 (51.6) | 66893 (30.4) | 62444 (28.3) | 22936 (10.4) | 94380 (42.8) | 125885 (57.2) |
| Jeonju | 107711 (48.4) | 114780 (51.6) | 64631 (29.0) | 64594 (29.0) | 24850 (11.2) | 95276 (42.8) | 127215 (57.2) |
| Jeonju | 108762 (48.4) | 115844 (51.6) | 62221 (27.7) | 66358 (29.5) | 27263 (12.1) | 96121 (42.8) | 128485 (57.2) |
| Jeonju | 109667 (48.4) | 116779 (51.6) | 59708 (26.4) | 68560 (30.3) | 29459 (13.0) | 96901 (42.8) | 129545 (57.2) |
| Jeonju | 110609 (48.5) | 117686 (51.5) | 57319 (25.1) | 70594 (30.9) | 31763 (13.9) | 97704 (42.8) | 130591 (57.2) |
| Jeonju | 109792 (48.4) | 117002 (51.6) | 54824 (24.2) | 72201 (31.8) | 33098 (14.6) | 97045 (42.8) | 129749 (57.2) |
| Jeonju | 109026 (48.4) | 116318 (51.6) | 52554 (23.3) | 74409 (33.0) | 34011 (15.1) | 96444 (42.8) | 128900 (57.2) |
| Jeonju | 108346 (48.4) | 115683 (51.6) | 50928 (22.7) | 75721 (33.8) | 35551 (15.9) | 95865 (42.8) | 128164 (57.2) |
| Jeonju | 107619 (48.3) | 115066 (51.7) | 49547 (22.2) | 77235 (34.7) | 36822 (16.5) | 95301 (42.8) | 127384 (57.2) |
| Jeonju | 106888 (48.3) | 114444 (51.7) | 48690 (22.0) | 78113 (35.3) | 38609 (17.4) | 94745 (42.8) | 126587 (57.2) |
| Pohang | 43984 (50.7) | 42843 (49.3) | 25926 (29.9) | 30048 (34.6) | 8936 (10.3) | 29757 (34.3) | 57070 (65.7) |
| Pohang | 44430 (50.7) | 43262 (49.3) | 24986 (28.5) | 30978 (35.3) | 9628 (11.0) | 29990 (34.2) | 57702 (65.8) |
| Pohang | 44871 (50.7) | 43684 (49.3) | 23935 (27.0) | 31735 (35.8) | 10619 (12.0) | 30252 (34.2) | 58303 (65.8) |
| Pohang | 45268 (50.7) | 44073 (49.3) | 22714 (25.4) | 32566 (36.5) | 11664 (13.1) | 30482 (34.1) | 58859 (65.9) |
| Pohang | 45652 (50.7) | 44438 (49.3) | 21681 (24.1) | 33187 (36.8) | 12769 (14.2) | 30680 (34.1) | 59410 (65.9) |
| Pohang | 45321 (50.6) | 44176 (49.4) | 20647 (23.1) | 33660 (37.6) | 13367 (14.9) | 30413 (34.0) | 59084 (66.0) |
| Pohang | 44952 (50.6) | 43875 (49.4) | 19727 (22.2) | 34005 (38.3) | 13998 (15.8) | 30107 (33.9) | 58720 (66.1) |
| Pohang | 44649 (50.6) | 43555 (49.4) | 19017 (21.6) | 33961 (38.5) | 14939 (16.9) | 29830 (33.8) | 58374 (66.2) |
| Pohang | 44366 (50.6) | 43295 (49.4) | 18339 (20.9) | 34050 (38.8) | 15854 (18.1) | 29600 (33.8) | 58061 (66.2) |
| Pohang | 44030 (50.6) | 43047 (49.4) | 17889 (20.5) | 33849 (38.9) | 16872 (19.4) | 29365 (33.7) | 57712 (66.3) |

Table S3. The difference between pooled estimates for gender stratified estimation

| Periods before and after  the 2016 Gyeongju earthquake | Pooled difference-in-difference estimates | | | Cochrane  Q statistics | p-value for  heterogeneity |
| --- | --- | --- | --- | --- | --- |
|  | Total | Men | Women |  |  |
| 2014.09-2015.08 | Ref | | |  |  |
| 2015.09-2016.08 | 0.97 (0.86, 1.11) | 0.86 (0.75, 0.98) | 1.13 (0.97, 1.31) | 7.36 | 0.01 |
| 2016.09-2017.08 | 1.58 (1.43, 1.73)^a^ | 1.38 (1.20, 1.60)^a^ | 1.85 (1.60, 2.13)^a^ | 9.49 | <0.01 |
| 2017.09-2018.08 | 1.33 (1.21, 1.46)^a^ | 1.30 (1.15, 1.47)^a^ | 1.38 (1.19, 1.60)^a^ | 0.37 | 0.54 |
| 2018.09-2019.08 | 1.15 (1.04, 1.27)^a^ | 1.10 (0.97, 1.24) | 1.21 (1.04, 1.41)^a^ | 0.95 | 0.33 |

^a^ P-value <0.05 for pooled difference-in-difference estimation

Table S4. The difference between pooled estimates for age group stratified estimation

| Periods before and after  the 2016 Gyeongju earthquake | Pooled difference-in-difference estimates | | | | Cochrane  Q statistics | p-value for  heterogeneity |
| --- | --- | --- | --- | --- | --- | --- |
|  | Total | 25-44 | 45-64 | 65- |  |  |
| 2014.09-2015.08 | Ref | | | |  |  |
| 2015.09-2016.08 | 0.97 (0.86, 1.11) | 0.98 (0.63, 1.52) | 1.00 (0.85, 1.17) | 0.98 (0.82, 1.17) | 0.16 | 0.69 |
| 2016.09-2017.08 | 1.58 (1.43, 1.73)a | 2.36 (1.50, 3.73)a | 1.65 (1.42, 1.91)a | 1.48 (1.25, 1.74)a | 3.92 | 0.05 |
| 2017.09-2018.08 | 1.33 (1.21, 1.46)a | 1.94 (1.27, 2.97)a | 1.31 (1.13, 1.53)a | 1.30 (1.10, 1.54)a | 3.12 | 0.08 |
| 2018.09-2019.08 | 1.15 (1.04, 1.27)a | 1.37 (0.88, 2.14) | 1.07 (0.91, 1.25) | 1.20 (1.00, 1.42)a | 1.66 | 0.2 |

^a^ P-value <0.05 for pooled difference-in-difference estimation

Table S5. The difference between pooled estimates for income group stratified estimation

| Periods before and after  the 2016 Gyeongju earthquake | Pooled difference-in-difference estimates | | | Cochrane  Q statistics | p-value for  heterogeneity |
| --- | --- | --- | --- | --- | --- |
|  | Total | Low-income | High-income |  |  |
| 2014.09-2015.08 | Ref | | |  |  |
| 2015.09-2016.08 | 0.97 (0.86, 1.11) | 1.03 (0.88, 1.21) | 0.93 (0.82, 1.06) | 0.93 | 0.33 |
| 2016.09-2017.08 | 1.58 (1.43, 1.73)a | 1.77 (1.52, 2.07)a | 1.47 (1.31, 1.65)a | 3.52 | 0.06 |
| 2017.09-2018.08 | 1.33 (1.21, 1.46)a | 1.25 (1.07, 1.46)a | 1.38 (1.23, 1.56)a | 0.96 | 0.33 |
| 2018.09-2019.08 | 1.15 (1.04, 1.27)a | 1.23 (1.05, 1.44)a | 1.10 (0.98, 1.25) | 1.15 | 0.28 |

^a^ P-value <0.05 for pooled difference-in-difference estimation

Table S6. Association between the Gyeongju earthquake and changes in the monthly incidence of ischemic heart disease patients in control cities.

|  | Monthly average incidence of IHD patients (N) | | | Age standardized average monthly incidence rate (/1,000,000 person) | | | Difference-in-difference estimates (95% CI) | |
| --- | --- | --- | --- | --- | --- | --- | --- | --- |
|  | Pohang | Gimpo | Jeonju | Pohang | Gimpo | Jeonju | Gimpo  vs Pohang | Gimpo  vs Jeonju |
| 2014.09-2015.08 | 34.1 | 38.6 | 68.8 | 211.2 | 225.4 | 189.8 |  |  |
| 2015.09-2016.08 | 30.6 | 39.0 | 76.9 | 194.3 | 223.3 | 206.7 | 1.12 (0.92, 1.36) | 0.90 (0.77, 1.05) |
| 2016.09-2017.08 | 29.9 | 37.2 | 70.5 | 179.6 | 196.8 | 184.2 | 1.09 (0.90, 1.33) | 0.93 (0.79, 1.09) |
| 2017.09-2018.08 | 32.8 | 39.6 | 66.0 | 190.9 | 212.8 | 167.6 | 1.06 (0.87, 1.28) | 1.05 (0.90, 1.24) |
| 2018.09-2019.08 | 30.0 | 37.8 | 62.3 | 169.2 | 199.6 | 153.3 | 1.10 (0.91, 1.34) | 1.06 (0.91, 1.25) |

Table S7. The difference-in-difference estimates with additional adjustment of monthly average temperature

|  | Difference-in-difference estimates  (95% confidence interval) | | | |
| --- | --- | --- | --- | --- |
|  | Gyeongju  vs Pohang | Gyeongju  vs Gimpo | Gyeongju  vs Jeonju | Pooled  estimates |
| Total |  |  |  |  |
| 2014.09-2015.08 |  |  |  |  |
| 2015.09-2016.08 | 1.10 (0.91, 1.33) | 0.98 (0.82, 1.17) | 0.88 (0.75, 1.02) | 0.97 (0.86, 1.10) |
| 2016.09-2017.08 | 1.73 (1.44, 2.07) | 1.60 (1.35, 1.89) | 1.48 (1.28, 1.71) | 1.58 (1.44, 1.74) |
| 2017.09-2018.08 | 1.35 (1.13, 1.62) | 1.29 (1.09, 1.53) | 1.36 (1.17, 1.57) | 1.34 (1.22, 1.47) |
| 2018.09-2019.08 | 1.19 (0.99, 1.44) | 1.10 (0.92, 1.31) | 1.17 (1.00, 1.36) | 1.15 (1.05, 1.27) |
